# Postoperative Changes in Systemic Immune Tolerance Following Major Oncologic versus Minor Maxillofacial Surgery

**DOI:** 10.3390/cancers15153755

**Published:** 2023-07-25

**Authors:** Leah Trumet, Jutta Ries, Philip Sobl, Niclas Ivenz, Falk Wehrhan, Rainer Lutz, Marco Kesting, Manuel Weber

**Affiliations:** 1 Department of Oral and Cranio-Maxillofacial Surgery, Uniklinikum Erlangen, Friedrich-Alexander-Universität Erlangen-Nürnberg (FAU), 91054 Erlangen, Germany; leah.trumet@icloud.com (L.T.); jutta.ries@uk-erlangen.de (J.R.);; 2 Deutsches Zentrum Immuntherapie (DZI), Comprehensive Cancer Center Erlangen-EMN (CCC ER-EMN), Friedrich-Alexander-Universität Erlangen-Nürnberg (FAU), 91054 Erlangen, Germany; 3 Department of Operative Dentistry and Periodontology, Friedrich-Alexander-Universität Erlangen-Nürnberg (FAU), 91054 Erlangen, Germany; 4 Private Office for Maxillofacial Surgery, 91781 Freiberg, Germany

**Keywords:** immune tolerance, IL-6, IL-10, FOXP3, PD-L1, immunotherapy, surgery

## Abstract

**Simple Summary:**

Surgical procedures can cause a systemic response of the immune system. This is relevant especially for multimodal tumor therapy in which immunotherapy plays an increasing role. How surgical interventions influence the systemic immune tolerance is not yet well understood. Aim of this study was to monitor changes in postoperative gene expression of cytokines and cellular immune markers in the blood of patients undergoing major maxillofacial tumor surgery compared to minor oral surgical procedures. It could be shown that there was a shift towards temporar systemic immunosuppression in the tumor surgery group compared to minor surgical procedures. The degree of immunosuppression correlated with the duration of surgery with longer procedures caused higher expression of immunosuppressive parameters. However, there were no signs for long-lasting immunosuppression as all analyzed markers approached baseline levels until post-op day 10. The results of this study might be relevant for better timing of neoadjuvant immunotherapy in the multimodal therapy of oral cancer and other malignancies.

**Abstract:**

Background: There is increasing evidence of the benefits of adjuvant and neoadjuvant immunotherapy in the treatment of solid malignancies like oral squamous cell carcinoma (OSCC). To optimize (neo-)adjuvant treatment, the systemic immunomodulatory effects of tumor surgery itself need to be considered. Currently, there is little evidence on the immunological effects of major surgery, such as free microvascular flap reconstruction. The current study aims to analyze how and to what extent maxillofacial surgery affects systemic parameters of immune tolerance. Methods: A total of 50 peripheral whole blood samples from patients (Group 1 (G1) = extensive OSCC surgery; Group 2 (G2) = free flap reconstruction without persistent malignant disease; Group 3 (G3) = minor maxillofacial surgery) undergoing surgery were included for real-time quantitative polymerase chain reaction (RT-qPCR) to examine changes in mRNA expression of the biomarkers IL-6, IL-10, FOXP3, and PD-L1. Blood samples were taken immediately before and after surgery as well as on the second, fourth, and tenth postoperative days. Differences in mRNA expression between groups and time points were calculated using statistical tests, including Mann–Whitney U-test and Pearson correlation analysis. Results: Comparing postoperative expression of G1 and G3, there was a significantly higher PD-L1 expression (*p* = 0.015) in G1 compared to G3 and a significantly lower IL-6 (*p* = 0.001) and FOXP3 (*p* = 0.016) expression. Interestingly, IL-10 expression was higher pre- (0.05) and postoperative (*p* < 0.001) in G1 compared to G3. Additionally, in G1, there was a significant overexpression of IL-10 post-surgery compared to the preoperative value (*p* = 0.03) and a downregulated expression of FOXP3 between pre- and 2 d post-surgery (*p* = 0.04). Furthermore, there was a significant correlation between the duration of surgery and the perioperative expression changes of the analyzed biomarkers. As the duration of surgery increased, the expression of IL-10 and PD-L1 increased, and the expression of IL-6 and FOXP3 decreased. Conclusion: Extensive surgery in OSCC patients is associated with a transient shift toward postoperative systemic immune tolerance compared with patients undergoing minor surgery. However, even extensive surgery causes no signs of long-lasting systemic immunosuppression. The degree of immune tolerance that occurred was associated with the duration of surgery. This supports efforts to minimize the duration of surgery.

## 1. Introduction

Surgery is an essential part of the treatment of many diseases, including a relevant number of cancer patients. However, these procedures also cause tissue trauma, which has an impact on the immune system. Until now, the alteration of the immune system that may result from surgical procedures has not been sufficiently investigated [1]. The immune response situation of patients is of special interest regarding the increasing use of immunotherapy in the treatment of solid malignancies, including oral cancer. Thereby, the application of immunotherapy using immune checkpoint inhibitors (ICIs) is shifting from the palliative setting to earlier disease stages [2]. Primary treatment of oral squamous cell carcinomas (OSCCs) involves invasive surgical procedures that go ahead with resection of the tumor, cervical lymph node dissection, and microvascular reconstruction involving free flap harvesting at a secondary site of the body. In addition, OSCC surgery is associated with relatively long durations due to microvascular anastomosis and the necessity of reconstruction in the spatially limited oral cavity. However, it is not yet understood in which temporal relation to a primary surgical therapy of OSCC and ICI therapy should be performed. Adjuvant, as well as neoadjuvant use of ICI, is discussed [3]. To better assess the timing of immunotherapy, an improved understanding of the effects of surgical procedures on the immunologic host response would be helpful in defining the point of time ICI therapy unfolds the best effect.

It is shown that patients receiving immunosuppressive corticosteroids simultaneous to ICI immunotherapy have a worse therapeutic response compared to cancer patients not receiving corticosteroids. However, even in an immunosuppressed patient cohort, immunotherapy achieved a therapeutic effect in a relevant proportion of individuals [4]. However, there are also cancer patients without any obvious immunosuppression not responding to ICI therapy at all [2].

There are several immunological changes in response to surgical procedures described, such as decreased antigen presentation and attenuated T-helper cell response. These changes are associated with immunosuppression and can negatively influence patient outcomes after surgery [1]. In particular, the temporal course of immunological changes in the perioperative period and the reversibility of these changes are not extensively understood [1].

During postoperative wound healing processes, increased levels of M2-polarized, tumor-promoting macrophages, myeloid-derived suppressor cells (MDSCs), regulatory T-cells (T_regs_), as well as increased expression of programmed cell death ligand 1 (PD-L1) is described [5]. These immunologic changes could open a window of opportunity for disseminated tumor cells to develop a local recurrence or metastasis [5]. Surgical tumor therapy is associated with an increase in circulating tumor cells [6]. In combination with local or systemic immunosuppression associated with surgical trauma, this could contribute to premetastatic niche formation and give rise to local recurrences or lymph nodes and distant metastases [5,6].

To obtain a better knowledge of the immunological shifts after surgery, we measured the mRNA expressions of the following biomarkers: Interleukin 6 (IL-6), Interleukin 10 (IL-10), Forkhead Box Protein 3 (FOXP3), and PD-L1 at different time points pre- and postoperative. In this analysis, we compared three groups of patients: (G1) OSCC patients receiving surgical tumor resection, cervical lymph node surgery (neck dissection), and microvascular reconstruction; (G2) patients without active malignancy and with extended microvascular reconstructive surgery and (G3) patients with minor maxillofacial surgery procedures. 

To distinguish between the expression course of different inflammation mediators, we investigated both pro- and anti-inflammatory interleukins and immune parameters. Previously, it was found that both the predominantly pro-inflammatory IL-6 and the anti-inflammatory IL-10 were found to be elevated in response to tissue damage [7].

IL-10 is expressed by T-effector cells, T_regs_, B-cells, antigen-presenting cells (APCs), and others [8]. In addition, IL-10 is produced by tumor cells [1,9]. FOXP3 is expressed in T_regs_ and in several tumor cells and is involved in multiple immunological functions [10,11,12]. T_regs_ influence the immune system through direct cell–cell interaction and cytokine secretion and are associated with immune tolerance [13]. T_regs_ inhibit maturation and function of APCs and trigger the release of cytokines, such as IL-10, which can induce immunosuppression by decreased activation of effector cells [14].

In many cancer types, increased numbers of FOXP3^+^ T_reg_ cells have also been shown to affect the efficacy of immunotherapies and overall survival and are associated with a higher risk of metastasis [10,14,15]. In recent studies, there were assumptions that T_regs,_ in general, could be responsible for a reduced response to ICI therapy [16]. T_reg_ cells can also express PD-L1, which is associated with a systemic state of immune tolerance [10,14,17,18].

PD-L1 is expressed by APCs and tumor cells and is involved in the OSCC immune escape mechanisms and highly relevant for ICI-based therapy against the PD-L1 receptor PD1 [18,19].

The aim of the present study was to analyze the expression of IL-6, IL-10, FOXP3, and PD-L1 in peripheral blood of maxillofacial surgery patients receiving different extends of surgery for malignant and non-malignant diseases at multiple time points up to 10 days postoperatively.

## 2. Materials and Methods

### 2.1. Patients Collective

For this study, peripheral whole blood samples from 50 patients aged between 19 and 80 years, who were treated at the Department of Oral and Cranio-Maxillofacial Surgery at Erlangen University Hospital, were analyzed. Enrollment into this study, from May 2019 to September 2021, was performed with the full consent of the patients and with ethics committee approval (application number 415_20 B). The samples were taken from individuals who were subdivided into three groups. Group 1 (G1) included 25 patients who underwent resection surgery for primary oral squamous cell carcinoma (OSCC) with selective supraomohyoideal neck dissection (ND) and microvascular free flap reconstruction, with no other malignancies present. The Group 2 (G2) patient cohort (*n* = 7) included microvascular free flap reconstructions of defects that were not due to malignant diseases. All patients in Group 3 (*n* = 18) underwent minor maxillofacial surgery like dentoalveolar surgery or trauma surgery and were healthy regarding malignant diseases. All patients were classified according to disease, sex, age, ASA classification, type, and duration of surgery. In ASA classification, patients are divided into 5 groups, and this is considered to be a description of the physical condition of the patients, taking into account their diseases [20]. In addition, the patients from G1 were categorized by TNM classification. A total of 25 patients, partly from G1 and partly from G2, did receive a blood transfusion during the collection of the samples included in this study.

All patients with oral cancer (G1) were treated according to the German treatment guidelines for oral squamous cell carcinomas [21]. This includes a tracheostomy at the beginning of the surgical procedure. This is followed by an ipsilateral neck dissection, including levels 1–3 after Robbins [22]. If the tumor did cross the midline, a bilateral neck dissection levels 1–3 after Robbins was carried out. If highly suspicious lymph nodes were detectable in level 2 or 3 on preoperative CT scans or during intra-operative frozen histologic sections, the neck dissection was extended to level 5 ipsilaterally and levels 1–3 contralaterally.

After completion of neck dissection, transoral tumor resection with security margins of at least 5 mm was performed. If the tumor had direct contact with the mandibular bone, a mandibular box resection or mandibular continuity resection was performed. Reconstruction was performed in all cases using microvascular tissue transfer. Tumor localization, resection extension, and method of reconstruction for all cases in Group 1 are provided in Supplementary Table S1.

The routine postoperative analgetic regime was ibuprofen 600 mg three times daily and metamizole 1000 mg four times daily in all groups. Low- and medium-potency opioids were applied if necessary.

### 2.2. Sampling of Peripheral Blood

From all patients, 2.5 mL of whole peripheral venous blood was collected in a PAXgene^®^ Blood miRNA Tube (PreAnalytiX GmbH, Hombrechtikon, Switzerland). These samples were obtained at five different time points: before surgery’s first cut (pre), after surgery’s last suture (post), at the second postoperative day (2 d post), at the fourth postoperative day (4 d post) and at the tenth postoperative day (10 d post). The samples were carefully inverted 8–10 times, incubated at room temperature for two hours, and frozen at −20 °C for 24 h before further processing.

### 2.3. Analysis of IL6, IL10, FOXP3, and PD-L1 Expression by Quantitative Real-Time Reverse-Transcriptase Polymerase Chain Reaction (RT-qPCR)

Further processing of the collected blood samples occurred in the biological laboratory of the Department of Oral and Cranio-Maxillofacial Surgery, Friedrich-Alexander-Universität Erlangen-Nürnberg (FAU). Using the NanoDrop 1000 spectrophotometer (ThermoFisher Scientific Company, Waltham, MA, USA) with wavelength 260 nm, the RNA concentration in the samples was determined. Only samples with RNA concentration >50 µg/mL were included in further analyses. Total RNA was transcribed into cDNA using the Transcriptor high-capacity kit (Applied Biosystems, Waltham, MA, USA) according to the manufacturer’s specifications. All samples were centrifuged and subjected to 50-cycle RT-qPCR in the QuantStudio 6 Pro Real-Time PCR System (Applied Biosystems, Waltham, MA, USA).

Different primers (Invitrogen, Carlsbad, CA, USA) and Power SYBR Green PCR mastermix (Thermo Fisher Scientific, Waltham, MA, USA) were used for the specific analysis of the expressions of IL6, IL10, FOXP3, and PD-L1. The exact primer length, base sequence, amplicon length, melting temperature ϑ, and accession number for each analyzed mRNA expression can be found in Table 1 GAPDH served as an endogenous control to normalize Ct values.

### 2.4. Statistical Analysis

The SPSS 23 statistical software package (SPSS Inc., Chicago, IL, USA) was used for statistical analysis based on the generated ΔCt data from RT-qPCR. The fold change (FC) was calculated using the ∆∆Ct method. An exploratory data analysis was performed on the collected data, and then, due to the absence of a normal distribution, the nonparametric Mann–Whitney U-test was used to determine whether expression levels differed significantly between the groups. In our analysis, a *p*-value ≤ 0.05 was considered statistically significant, and a *p*-value ≤ 0.001 was considered highly significant. In addition, the mean values of the experimental groups and the mRNA expression of IL6, IL10, Foxp3, and PD-L1 at specific time points and within groups were compared. Postoperative progression curves and Pearson correlations were also generated for each experimental group and biomarker. In addition, boxplots and scatterplots were made to depict the statistical findings.

## 3. Results

The ΔCt_pre_ value was used as a baseline to determine the magnitude of changes in expression levels (FC) and their significance (*p*-value) at different time points. Standard deviation (SD).

### 3.1. Patients Collective

In G1 (*n* = 25), 16% were female, and 84% male. The average patient in G1 was 62.75 ± 9.55 years old, and the average preoperative ASA score was 2.36 ± 0.62. The average duration of surgery was 567 ± 147 min, which was due to the complex tumor resection and reconstruction cases. Out of seven patients in G2 (*n* = 7), 14% were female and 86% male, with an average ASA score of 2.43 ± 0.49 and an average of 478 ± 123 min per surgery. In contrast to G1 and G2, the proportion of women in G3 (*n* = 18) was almost as high as that of men, at 44%. The mean age was 49.11 ± 19.72 years. The preoperative ASA score in G3 was 1.44 ± 0.62, and the average duration of surgery was 52 ± 29 min.

### 3.2. IL-6

**Figure 1:**
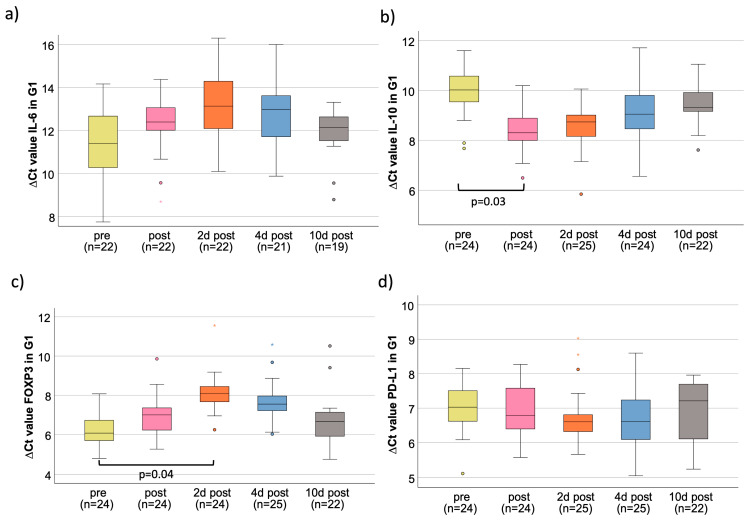
Perioperative time courses for G1 for all four markers investigated. Box plots showing the median expression rates of IL-6 (**a**), IL-10 (**b**), FOXP3 (**c**), and PD-L1 (**d**) in peripheral whole blood samples at five different time points (tp) of G1 patients. The time points were pre-, post-, 2 d post-, 4 d post-, and 10 d post-tumor resection surgery. Values represent the number of cases (*n*), ΔCt values (higher ∆Ct values indicate a lower expression) at the different tp. (**a**) Perioperative IL-6 mRNA expression; (**b**) perioperative IL-10 mRNA expression; (**c**) perioperative FOXP3 mRNA expression; (**d**) perioperative PD-L1 mRNA expression. °,* outliners.

In G1, the median ΔCt_pre_ value was 11.39 and increased post-surgery to a maximum of on the second post-op day (ΔCt_2dpost_ = 13.14) and then normalized during the post-operative course with no statistically significant expression changes (Table 2, Figure 1a). In G2, the median ΔCt_pre_ value was 11.35. The median value increased slightly but not significantly until postoperative day 2 (ΔCt_2dpost_ = 13.51) and then approached the starting expression again, with a decrease in expression (ΔCt_10dpost_ = 12.19) on the 10th postoperative day. In G3, the median ΔCt_pre_ value was 11.03. In summary, there was no significant expression change but an increase in the median ΔCt value (Table 2).

**Figure 2:**
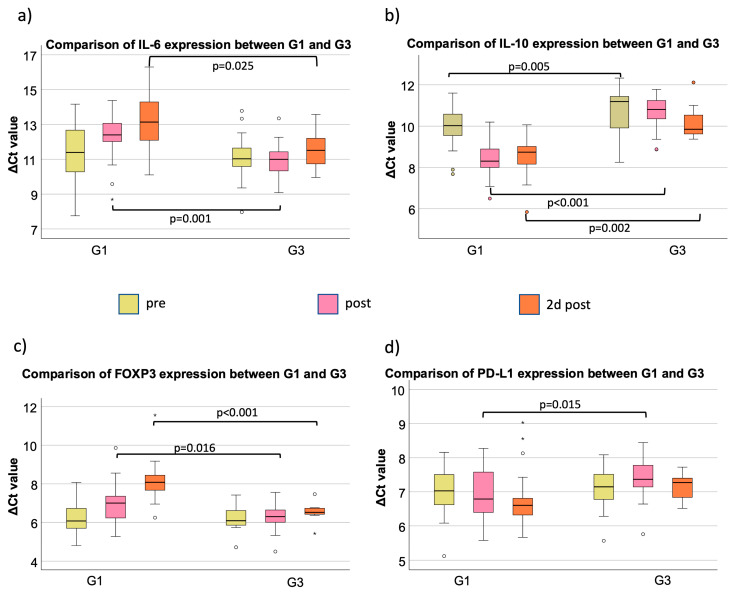
Comparison of marker expression on three perioperative time points (pre, post, and 2 d post) between G1 and G3. Boxplots showing the expression comparison of different markers ((IL-6 (**a**), IL-10 (**b**), FOXP3 (**c**), and PD-L1 (**d**)) between groups G1 and G3 at different time points ((pre (yellow), post (pink), and 2 d post (orange)). The significant *p*-values are integrated into the boxplots. (**a**) Perioperative IL-6 mRNA expression; (**b**) perioperative IL-10 mRNA expression; (**c**) perioperative FOXP3 mRNA expression; (**d**) perioperative PD-L1 mRNA expression. °,* outliners.

**Figure 3:**
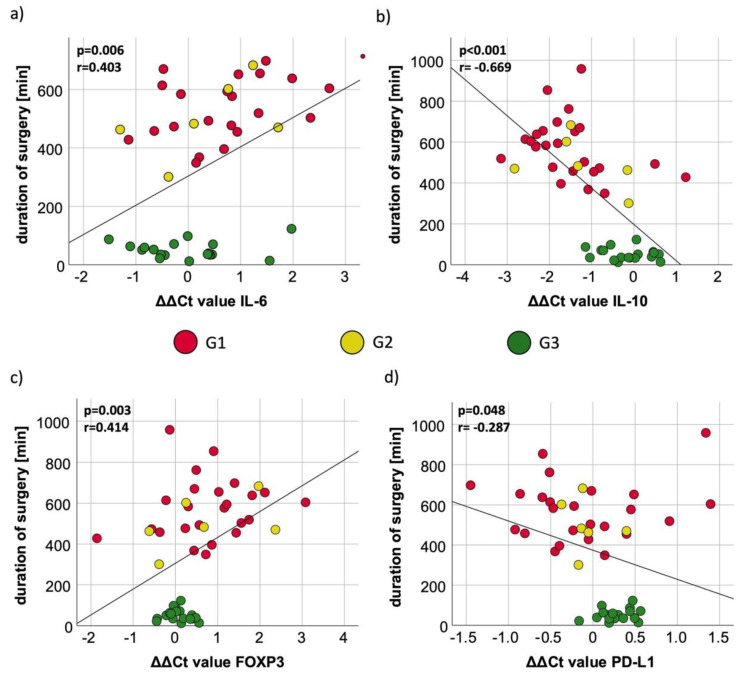
Correlation between duration of surgery and marker expression. The figures show the correlation between marker expression of IL-6 (**a**), IL-10 (**b**), FOXP3 (**c**), and PD-L1 (**d**) and the duration of the surgery for G1, G2, and G3. The measure of linear correlation was made doing the Pearson correlation test. The duration of surgery was measured in minutes (min), and the expression was measured using the ΔΔCt value, which was generated by subtracting the ΔCt_post_ from ΔCt_pre_ value. The significant *p*-values are written in bold letters. r: Pearson correlation coefficient; (**a**) IL-6: r_IL-6_ = 0.403; p_IL-6_ = 0.006; (**b**) IL-10: r_IL-10_ = −0.669; p_IL-10_ < 0.001; (**c**) FOXP3: r_FOXP3_ = 0.414; p_FOXP3_ = 0.003; (**d**) PD-L1: r_PD-L1_ = −0.287; p_PD-L1_ = 0.048.

IL-6 expression post differs highly statistically significant between G1 and G3 (*p* < 0.01); 2 days post is also statistically significant between G1 and G3 (*p* = 0.025) (Figure 2a, Table 3. In addition, an expression difference on post and 2 d post could be shown between G2 and G3 (*p* = 0.02) (Table 3). Pearson correlation analysis revealed a significant positive correlation between the duration of surgery and the IL-6 ΔΔCt value (ΔCt (post-op) −ΔCt (pre-op) (*p* = 0.006; Figure 3a). In Groups 1 and 2, longer surgery time was associated with decreased IL-6 expression. The measurements of minor surgical procedures (G3) showed a relatively wide spreading with low expression changes (Figure 3a).

### 3.3. IL-10

In G1, the median ΔCt_pre_ value was 10.02. The ΔCt value decreased significantly directly after surgery, which means a significant increase in the expression of IL-10 (p_post_ = 0.03; FC_post_ = 3.27). In the postoperative course, first, the previously described increase in expression and then an approximation to the starting value was observed without reaching statistical significance (Table 4, Figure 1b). In G2, the median ΔCt_pre_ value was 9.98. The postoperative course showed a slight increase in expression until postoperative day 2, and a decrease was measured on 4th and 10th postoperative days with almost the same values on day 10 as before surgery. In G3, there was no significant change in expression in the pre- and postoperative course. The highest measured fold change to “pre” (FC_2dpost_ = 2.53) was on the second postoperative day (Table 4). The IL-10 expression between G1 and G3 was highly statistically significant on pre-, post-, and 2 d post-surgery (*p* < 0.01) (Figure 2b, Table 3). The comparison of expression from G3 to G2 was highly statistically significant, modified directly post- and 2 d post-surgery (*p* < 0.01), whereas there was no statistical relevance of different expressions comparing G2 to G1 (Table 3). The duration of surgery had a significant influence on the expression of IL-10 as the correlation of the procedure time and ΔΔCt value correlated strongly in a negative manner, which indicates an increase in IL-10 expression per time operated (*p* < 0.001; r = −0.669;) (Figure 3b). G3 showed the lowest changes in IL-10 expression. G1 and G2 showed a higher span of operated minutes with clear visibility of the association of higher procedure time with increased IL-10 expression (Figure 3b).

### 3.4. FOXP3

G1 showed a median ΔCt_pre_ value of 6.07. The expression of FOXP3 decreased significantly on the second day during the postoperative course (p_2dpost_ = 0.04; FC_2dpost_ = 0.25). On the 10th postoperative day, the FOXP3 expression was close to the first sampling with a median ΔCt_10d_ value of 6.67 (p_10d_ = 0.27) (Table 5). All in all, an expression decrease after surgery, with a peak on the second post-op day, followed by normalization of expression to the pre-op situation, could be shown (Figure 1c). In G2, the median ΔCt_pre_ value was 6.24. In summary, there was a peak on postoperative day 2, with no significance between the timeslots. The median preoperative ΔCt_pre_ value in G3 was 6.09. In the early postoperative course, there was a slight decrease in expression, which stopped on the fourth day after surgery, where there was a small increase with a ΔCt_4dpost_ value of 5.96 (p_4dpost_ = 0.30) (Table 5). FOXP3 initially shows no expression differences among the three different groups (p_preG2toG1_ = 0.37; p_preG3toG1_ = 0.98). The comparison of G2 to G1, G3 to G1, and G3 to G2 only showed expression differences after surgery (Table 3). Directly after surgery, there was a higher FOXP3 expression in G3 compared to G1 (p_post_ = 0.02) (Figure 2c, Table 3). A total of 2 days after surgery, the expression in G3 was still overexpressed compared to G1 (*p* < 0.01) and G2 (*p* = 0.02) (Table 3). A prolonged invasive surgical procedure, which was especially performed in G1 and G2, was associated with a decreased expression of FOXP3 in peripheral blood (*p* = 0.003) (Figure 3c). The surgical procedures in G3 did have less variety in duration and expression, and therefore, a close spreading around the starting value is observable (Figure 3c).

### 3.5. PD-L1

The median ΔCt_pre_ value was 7.03 in G1. There were minor expression changes the days after surgery; however, they were never statistically significant (Table 6, Figure 1d). In G2, the median ΔCt_pre_ value was 7.45, which showed almost no changes in the course. The same situation occurred for G3, but the expression of PD-L1 was slightly increased on the fourth postoperative day but did not reach statistical relevance (p_4dpost_ = 0.49) (Table 6). The marker expression showed no statistically significant differences pre-surgery between G1, G2, and G3 (Table 3). But, post and 2 d post, there was less expression of PD-L1 in G3 compared to G1 (p_post_ = 0.02; p_2dpost_ = 0.053) (Figure 2d, Table 3). Compared to the accumulation around the ΔΔCt value of 0 with almost no duration change in G3, the PD-L1 expression and duration of surgery in G1 and G2 showed more variability (Figure 3d). Analyzing the ΔΔCt values of PD-L1 in all groups, there was a significant association between increased time of surgery with increased expression levels of PD-L1 (Figure 3d).

## 4. Discussion

### 4.1. Influence of Surgical Trauma on Peripheral Immune Tolerance

When the immune system is activated under various conditions, for example, by injury, infectious disease, or surgery, IL-6 levels increase due to its production by monocytes and macrophages [23,24]. IL-6 acts initially pro-inflammatory and can act anti-inflammatory in the later course of the immune response [7,24]. Even though IL-6 is mainly a pro-inflammatory cytokine, it can recruit MDSC cells to the tumor site and therefore promote the tumor immune escape [5]. 

In the OSCC patient group (G1), in peripheral blood, a decrease in IL-6 mRNA expression could be shown with the lowest levels measured on postoperative day 2 and then an attenuation upon reaching levels close to the preoperative value on day 10. However, none of the expression changes in relation to the preoperative value reached statistical significance.

Comparing G1, G2 (oral microvascular surgery without head-and-neck malignancy), and G3 (minor maxillofacial surgical procedures), IL-6 expression was significantly higher in G3 directly postoperatively as well as on day 2 compared to both G1 and G2. This indicates that patients receiving extensive microvascular head-and-neck surgery show lower postoperative IL-6 levels compared to minor surgical procedures.

In contrast to IL-6, the anti-inflammatory cytokine IL-10 showed a significant increase in expression directly postoperatively in OSCC patients (G1). Compared to G3, OSCC patients had increased IL-10 levels directly postoperatively as well as on day 2. The IL-10 expression course in G2 was similar compared to G1. Directly post-op as well as on day 2, IL-10 expression in G2 was significantly higher compared to G3. This indicates that the temporary elevation in peripheral IL-10 expression might be caused by the more extensive surgery in G1 and G2. 

In addition, the preoperative IL-10 expression in OSCC patients was significantly higher than in the control group G3. This finding indicates that OSCC patients might have a preexisting increase in IL-10, which may be caused by the malignant disease itself.

In previous studies by our research group, we have shown that surgical trauma has a potential negative impact on tumor progression that might be mediated by macrophages. Biopsy-induced tissue trauma was associated with a significant shift of macrophage polarization from the anti-tumoral M1 type to tumor-promoting M2 macrophages at the local tumor site [25]. The increased IL-10 expression and decreased IL-6 expression observed in the current study could be an indication that there is also a systemic shift toward M2 polarization in response to surgery. However, it needs to be noted that the systemic effects seem to be temporarily limited.

The expression of FOXP3 was significantly downregulated in the OSCC group on postoperative day 2 compared to the preoperative level. In the later postoperative course until day 10, the FOXP3 expression increased again, almost reaching the preoperative level. FOXP3-expressing T_reg_ cells are a highly immunosuppressive CD4^+^ T-cell population that migrate into inflammatory sites and tumors and suppress the activity of effector lymphocytes [5]. The decrease in peripheral blood FOXP3 expression indicates a decrease in peripheral T_reg_ cells. In breast cancer patients receiving mastectomy, initially on post-op day 1, a significant decrease in FOXP3 mRNA in the peripheral blood could be detected. However, on postoperative day 7, a significant increase in FOXP3 mRNA expression as well as flow-cytometric T-cell count was observed [26]. The FOXP3 decrease described on day 1 after mastectomy is consistent with the reduced expression we could detect on day 2. A consequent increase in FOXP3 expression above the preoperative level could not be reproduced by the current analysis in OSCC surgery. A hypothetical explanation could be the fact that breast cancer surgery is carried out at a completely sterile surgical site. In contrast, there are fresh wounds in the highly bacterially contaminated oral cavity, which could provide a pro-inflammatory stimulus.

Comparing the oral cancer surgery group (G1) with patients receiving minor maxillofacial surgery (G3), a significantly lower FOXP3 expression could be detected directly postoperatively as well as on day 2. Analyzing the patient cohort receiving microvascular oral reconstruction without head-and-neck malignancy (G2), a similar temporal course of FOXP3 expression compared to G1 can be observed. This indicates that the temporary decrease in FOXP3 expression in the early postoperative period is associated with major reconstructive surgery and is not present in minor cases.

The current analysis revealed a slight postoperative increase in PD-L1 expression in the peripheral blood without statistical significance that was attenuated until postoperative day 10 in the OSCC cancer group (G1). Comparing G1 and G3, the OSCC patients of G1 showed a significantly higher PD-L1 expression directly postoperatively and on day 2 compared to cases with minor surgery. In a breast cancer mouse model, macrophages, not tumor cells, were the main source of PD-L1 expression. PD-L1 expression was promoting tumor growth that could be inhibited by anti-PD1 therapy [27]. 

Our current data indicate that there is a systemic increase in PD-L1 expression in OSCC patients that are treated by surgery. However, this increase seems to be temporarily limited in the immediate postoperative period and seems to be not permanent. This finding is interesting as we could previously detect that initial preoperative peripheral blood PD-L1 expression in OSCC patients is associated with the occurrence of lymph node metastases and with patients’ survival [18,28].

It is postulated that surgery promotes inhibitory cytokines that lead to an immunosuppressive environment in potential areas of residual disease and foster quick disease progression [29]. In a mouse model, subtotal tumor resection was shown to increase levels of IL-1, IL-6, and IL-10. In addition, an increased M2 polarization of macrophages in the residual tumor mass, as well as an increased T_reg_ infiltration, was observed [29,30]. This is consistent with the previously described increase in M2 polarization in human OSCC tissue in association with biopsy-induced tissue trauma [25]. Those data have shown similar local immunological changes as we could systemically detect in our current study.

In an analysis of systemic IL-10 expression following major abdominal surgery, the highest IL-10 levels were detected two hours after the surgery [1]. Afterward, IL-10 mRNA and protein expression decreased again until 48 h after surgery [1]. This is consistent with the results of the current analysis. It is hypothesized that IL-10 leads to an impaired monocyte effector function associated with decreased HLA-DR expression. In addition, it was found that FOXP3 expression was decreased after abdominal surgery [1], which is a comparable expression pattern to our current data in OSCC patients undergoing major resective and reconstructive surgery.

In a flow-cytometric analysis of peripheral immune status on 10 patients receiving major abdominal surgery, postoperative alterations of innate immune cells were recovered until the second postoperative day [31]. However, alterations in T-cell numbers did not fully recover until postoperative day 5 [31]. The data from the current study also suggest that most systemic postoperative immune changes recover rapidly in the postoperative course.

As all analyzed biomarkers in the current study approximate the preoperative levels until postoperative day 10, one could hypothesize that there might be no long-lasting negative systemic immunologic effects of major maxillofacial surgery. However, there is evidence that cytokine expression in the immediate perioperative period is relevant for cancer prognosis [32]. In colorectal cancer patients, the application of IL-2 three days prior to surgery was able to improve overall survival in a phase II study [32]. Similar effects could also be shown in renal cancer in response to preoperative IL-2 application [32]. This indicates that even short alterations in systemic immune tolerance in the perioperative period might have an influence on long-term disease prognosis.

Besides cytokine therapy, the neoadjuvant use of immune checkpoint inhibitors could overcome direct perioperative, surgery-associated immune tolerance [32]. There are several studies showing increased survival rates if ICI treatment was applied prior to OSCC treatment [2]. Neoadjuvant treatment with anti-CTLA4 of advanced melanoma was associated with increased levels of circulating FOXP3-positive T_reg_ cells, which interestingly correlated with a better prognosis [33]. This indicates that the decreased levels of FOXP3 expression observed postoperatively in oral cancer patients compared to controls with minor surgery in the current study might also be an expression of impaired immune responsiveness.

### 4.2. Influence of Duration of Surgery on Peripheral Immune Tolerance

Comparing the preoperative and postoperative expression of the analyzed biomarkers (ΔΔCt values), we could assess the association between the increase or decrease in marker expression with the duration of surgery. We could detect a significant association between increased duration of surgery with decreased IL-6 and increased IL-10 expression. In addition, FOXP3 was significantly decreased, and PD-L1 significantly increased with longer durations of surgery.

In patients with esophageal cancer receiving surgical esophagectomy, on postoperative day 1, an increase in IL-6 and IL-8 expression was detected, which attenuated in the further postoperative course [34]. A longer operative time, as well as an open approach versus an endoscopic approach, were associated with a significant increase in IL-6 and IL-8 expression [34]. These data on postoperative IL-6 expression are consistent with the results of the current study indicating an association of perioperative increase in IL-6 expression with the duration of surgery and possibly the amount of surgical tissue trauma.

The observed PD-L1 increase with a longer duration of surgery could also be a consequence of increased immune tolerance, as peripheral blood PD-L1 expression has been negatively associated with patient survival [18,28].

### 4.3. Limitations of the Study

This study has some limitations that could be addressed in future research. G2 is limited to seven patients, so this group has less power than the other two groups.

Another limitation is that the effect of anesthesia cannot be identified exactly. The potential influence of anesthesia on the immune system is described and discussed elsewhere [35]. However, in the current study, all patients received comparable routine general anesthesiologic protocols, and therefore, a comparison between G1, G2, and G3 is possible. Also, an isolated investigation of invasiveness and duration of surgery is only possible to a limited point since more invasive surgeries also take longer on average. In addition, the effect of drugs, as well as blood transfusions given pre- and postoperatively, could not be discriminated separately. In this regard, patients in Groups 1 and 2 routinely have more home medications and require a larger number of perioperative drugs. This could also influence systemic immune parameters besides the surgical trauma itself. 

However, for our study, it was important to obtain an overview and a better understanding of immunological changes post-surgery for different surgical procedures presented by a consecutive group of patients under routine clinical conditions.

## 5. Conclusions

Major maxillofacial surgery procedures are associated with a short period of immediate perioperative systemic immunosuppression that quickly attenuates and is no longer detectable on the 10th postoperative day. Hence, there seems to be no long-lasting systemic immunosuppressive effect induced by major surgery. Neoadjuvant immunotherapy might be beneficial in protecting against immediate perioperative immunosuppression. Increased duration of surgery is associated with increased levels of perioperative immune tolerance. Therefore, it might be advantageous to keep the duration of surgery as short as possible also in complex oncological procedures.

Increased preoperative levels of IL-10 in OSCC patients may be an expression of a disease-related preexisting systemic immune tolerance.

## Figures and Tables

**Table 1:** Primer for RT-qPCR.

Real-Time qPCR					
Primer	Sequence (5′ to 3′)	Primer Length (bp)	Amplicon Length (bp)	Annealing Temp. (°C)	Accession Number
**IL-6/s**	TCG GTC CAG TTG CCT TCT CC	20	127	60	NM_000600
**IL-6/as**	TCT GAA GAG GTC AGT GGC TGT C	22	127	60	
**IL-10/s**	AAG GCG CAT GTG AAC TCC C	19	99	60	NM_000572.3
**IL10/as**	GGC CTT GCT CTT GTT TTC ACA G	22	99	61	
**FOXP3/s**	CAC TGC TGG CAA ATG GTG TC	20	226	60	NM_014009.4
**FOXP3/as**	TCA TCC AGA AGA TGG TCC GC	20	226	60	
**PD-L1/s**	AGC TAT GGT GGT GCC GAC TA	20	152	61	NM_014143.3 NM_001314029
**PD-L1/as**	CAG ATG ACT TCG GCC TTG GG	20	152	61	
**GAPDH/s**	GAC CCC TTC ATT GAC CTC AAC TA	23	122	60	NM_002046.5
**GAPDH/as**	GAA TTT GCC ATG GGT GGA AT	20	122	60	

The table shows the primers we selected for RT-qPCR mRNA expression analysis of IL-6, IL-10, FOXP3, and PD-L1. Also, the primer for the GAPDH as internal control is provided.

**Table 2:** Parameters of perioperative time courses for G1, G2, and G3 of IL-6.

IL6 in G1	Pre	Post	2 d Post	4 d Post	10 d Post
Sample size (*n*)	22	22	22	21	19
Median ΔCt value	11.39	12.40	13.14	12.94	12.15
Fold change to “pre“	1.00	0.50	0.30	0.34	0.59
Average (±standard derivation)	11.39 (±1.57)	12.32 (±1.41)	13.17 (±1.70)	12.80 (±1.69)	11.92 (±1.15)
*p*-value to “pre“	n.d.	0.20	0.19	0.07	0.19
**IL6 in G2**	**Pre**	**Post**	**2 d Post**	**4 d Post**	**10 d Post**
Sample size (*n*)	7	6	7	5	5
Median ΔCt value	11.35	11.76	13.51	11.55	12.19
Fold change to “pre“	1.00	0.75	0.22	0.87	0.56
Average (±standard derivation)	11.69 (±1.04)	12.15 (±1.11)	13.30 (±0.96)	11.71 (±1.06)	11.97 (±0.60)
*p*-value to “pre“	n.d.	0.35	0.12	0.43	0.49
**IL6 in G3**	**Pre**	**Post**	**2 d Post**	**4 d Post**	
Sample size (*n*)	18	18	7	5	
Median ΔCt value	11.03	11.00	11.51	12.37	
Fold change to “pre“	1.00	1.02	0.72	0.40	
Average (±standard derivation)	11.10 (±1.36)	11.04 (±1.02)	11.56 (±1.29)	12.15 (±0.89)	
*p*-value to “pre“	n.d.	0.33	0.44	0.31	

The table shows peripheral blood IL-6 mRNA expression of the pre- and postoperative time course. For each group (G1, G2, and G3) and time point (pre, post, 2 d post, 4 d post, and 10 d post), sample size (*n*), median ΔCt value, fold change (FC) to “pre”, average, and *p*-value to “pre” are provided. The FC was determined by the ΔΔCt method comparing the average ΔCt values of the different time points, starting from the pre-op value. The significant *p*-values are written in bold letters. G1: Primary tumor resection, microvascular free flap reconstruction, and selective neck dissection due to oral squamous cell carcinoma. G2: Microvascular free flap reconstruction without present malignancy. G3: Minor maxillofacial surgeries. n.d.: not determined.

**Table 3:** Comparison of marker expression between G1, G2, and G3.

IL6	Pre			Post			2 d Post		
	G1	G2	G3	G1	G2	G3	G1	G2	G3
Median ΔCt value	11.39	11.35	11.03	12.40	11.76	11.00	13.14	13.51	11.51
Fold change to G1	1.00	1.03	1.28	1.00	1.56	2.64	1.00	0.77	3.10
*p*-value to G1	n.d.	0.92	0.45	n.d.	0.22	**<0.01**	n.d.	0.80	**0.03**
*p*-value G2/G3	n.d.	0.36		n.d.	**0.02**		n.d.	**0.02**	
**IL10**	**Pre**			**Post**			**2 d Post**		
	**G1**	**G2**	**G3**	**G1**	**G2**	**G3**	**G1**	**G2**	**G3**
Median ΔCt value	10.02	9.98	11.19	8.31	8.90	10.81	8.74	8.88	9.85
Fold change to G1	1.00	1.03	0.44	1.00	0.66	0.18	1.00	0.91	0.46
*p*-value to G1	n.d.	0.89	**<0.01**	n.d.	0.21	**<0.01**	n.d.	0.19	**<0.01**
*p*-value G2/G3	n.d.	0.09		n.d.	**<0.01**		n.d.	**<0.01**	
**FOXP3**	**Pre**			**Post**			**2 d Post**		
	**G1**	**G2**	**G3**	**G1**	**G2**	**G3**	**G1**	**G2**	**G3**
Median ΔCt value	6.07	6.24	6.09	7.00	7.00	6.30	8.08	8.19	6.52
Fold change to G1	1.00	0.89	0.99	1.00	1.00	1.62	1.00	0.93	2.95
*p*-value to G1	n.d.	0.37	0.98	n.d.	0.80	**0.02**	n.d.	0.64	**<0.01**
*p*-value G2/G3	n.d.	0.40		n.d.	0.16		n.d.	**0.02**	
**PD-L1**	**Pre**			**Post**			**2 d Post**		
	**G1**	**G2**	**G3**	**G1**	**G2**	**G3**	**G1**	**G2**	**G3**
Median ΔCt value	7.03	7.45	7.15	6.79	7.21	7.37	6.60	7.19	7.27
Fold change to G1	1.00	0.75	0.92	1.00	0.75	0.67	1.00	0.66	0.63
*p*-value to G1	n.d.	0.32	0.70	n.d.	0.35	**0.02**	n.d.	0.08	0.05
*p*-value G2/G3	n.d.	0.51		n.d.	0.46		n.d.	0.95	

The table shows the comparison of G1, G2, and G3 of the peripheral blood mRNA expression of IL-6, IL-10, FOXP3, and PD-L1 of the pre- and post- and 2 d postoperative time course. For the analysis, sample size (*n*), median ΔCt value, fold change (FC) to “pre”, average, and *p*-value to “pre” are provided. The FC was determined by the ΔΔCt method comparing the average ΔCt values of the different time points, starting from the pre-op value. The significant *p*-values are written in bold letters. G1: Primary tumor resection, microvascular free flap reconstruction, and selective neck dissection due to oral squamous cell carcinoma. G2: Microvascular free flap reconstruction without present malignancy. G3: Minor maxillofacial surgeries. n.d.: not determined.

**Table 4:** Parameters of perioperative time courses for G1, G2, and G3 of IL-10.

IL10 in G1	Pre	Post	2 d Post	4 d Post	10 d Post
Sample size (*n*)	24	24	25	24	22
Median ΔCt value	10.02	8.31	8.74	9.05	9.31
Fold change to “pre“	1.00	3.27	2.43	1.96	1.64
Average (±standard derivation)	9.95 (±0.91)	8.45 (±0.84)	8.63 (±0.94)	9.18 (±1.08)	9.46 (±0.80)
*p*-value to “pre“	n.d.	**0.03**	0.06	0.15	0.08
**IL10 in G2**	**Pre**	**Post**	**2 d Post**	**4 d Post**	**10 d Post**
Sample size (*n*)	7	6	7	7	6
Median ΔCt value	9.98	8.90	8.88	9.21	9.97
Fold change to “pre“	1.00	2.11	2.14	1.71	1.01
Average (±standard derivation)	10.11 (±0.70)	8.88 (±0.62)	9.03 (±0.32)	9.36 (±0.50)	10.20 (±0.80)
*p*-value to “pre“	n.d.	0.33	0.28	0.21	0.48
**IL10 in G3**	**Pre**	**Post**	**2 d Post**	**4 d Post**	
Sample size (*n*)	18	18	7	5	
Median ΔCt value	11.19	10.81	9.85	10.67	
Fold change to “pre“	1.00	1.30	2.53	1.43	
Average (±standard derivation)	10.80 (±1.05)	10.64 (±0.85)	10.23 (±0.99)	10.81 (±0.51)	
*p*-value to “pre“	n.d.	0.48	0.26	0.39	

The table shows peripheral blood IL-10 mRNA expression of the pre- and postoperative time course. For each group (G1, G2, and G3) and time point (pre, post, 2 d post, 4 d post, and 10 d post), sample size (*n*), median ΔCt value, fold change (FC) to “pre”, average, and *p*-value to “pre” are provided. The FC was determined by the ΔΔCt method comparing the average ΔCt values of the different time points, starting from the pre-op value. The significant *p*-values are written in bold letters. G1: Primary tumor resection, microvascular free flap reconstruction, and selective neck dissection due to oral squamous cell carcinoma. G2: Microvascular free flap reconstruction without present malignancy. G3: Minor maxillofacial surgeries. n.d.: not determined.

**Table 5:** Parameters of perioperative time courses for G1, G2, and G3 of FOXP3.

FOXP3 in G1	Pre	Post	2 d Post	4 d Post	10 d Post
Sample size (*n*)	24	24	24	25	22
Median ΔCt value	6.07	7.00	8.08	7.55	6.67
Fold change to “pre“	1.00	0.52	0.25	0.36	0.66
Average (±standard derivation)	6.25 (±0.85)	7.01 (±1.05)	8.12 (±1.00)	7.74 (±1.21)	6.71 (±1.29)
*p*-value to “pre“	n.d.	0.27	**0.04**	0.06	0.27
**FOXP3 in G2**	**Pre**	**Post**	**2 d Post**	**4 d Post**	**10 d Post**
Sample size (*n*)	7	6	7	7	6
Median ΔCt value	6.24	7.00	8.19	7.66	6.79
Fold change to “pre“	1.00	0.59	0.26	0.37	0.68
Average (±standard derivation)	6.43 (±0.98)	7.17 (±1.33)	8.15 (±1.01)	7.69 (±1.29)	6.70 (±0.71)
*p*-value to “pre“	n.d.	0.33	0.18	0.25	0.39
**FOXP3 in G3**	**Pre**	**Post**	**2 d Post**	**4 d Post**	
Sample size (*n*)	18	18	7	5	
Median ΔCt value	6.09	6.30	6.52	5.96	
Fold change to “pre“	1.00	0.86	0.74	1.09	
Average (±standard derivation)	6.18 (±0.61)	6.25 (±0.67)	6.53 (±0.61)	5.96 (±0.69)	
*p*-value to “pre“	n.d.	0.39	0.46	0.30	

The table shows peripheral blood FOXP3 mRNA expression of the pre- and postoperative time course. For each group (G1, G2, and G3) and time point (pre, post, 2 d post, 4 d post, and 10 d post), sample size (*n*), median ΔCt value, fold change (FC) to “pre”, average, and *p*-value to “pre” are provided. The FC was determined by the ΔΔCt method comparing the average ΔCt values of the different time points, starting from the pre-op value. The significant *p*-values are written in bold letters. G1: Primary tumor resection, microvascular free flap reconstruction, and selective neck dissection due to oral squamous cell carcinoma. G2: Microvascular free flap reconstruction without present malignancy. G3: Minor maxillofacial surgeries. n.d.: not determined.

**Table 6:** Parameters of perioperative time courses for G1, G2, and G3 of PD-L1.

PD-L1 in G1	Pre	Post	2 d Post	4 d Post	10 d Post
Sample size (*n*)	24	24	25	25	22
Median ΔCt value	7.03	6.79	6.60	6.61	7.21
Fold change to “pre“	1.00	1.18	1.35	1.34	0.88
Average (±standard derivation)	7.00 (±0.68)	6.88 (±0.72)	6.76 (±0.80)	6.65 (±0.82)	6.87 (±0.96)
*p*-value to “pre“	n.d.	0.44	0.21	0.26	0.50
**PD-L1 in G2**	**Pre**	**Post**	**2 d Post**	**4 d Post**	**10 d Post**
Sample size (*n*)	7	6	7	7	6
Median ΔCt value	7.45	7.21	7.19	7.30	7.11
Fold change to “pre“	1.00	1.18	1.20	1.11	1.27
Average (±standard derivation)	7.23 (±0.63)	7.12 (±0.70)	7.14 (±0.56)	7.14 (±0.66)	6.69 (±1.93)
*p*-value to “pre“	n.d.	0.38	0.44	0.44	0.48
**PD-L1 in G3**	**Pre**	**Post**	**2 d Post**	**4 d Post**	
Sample size (*n*)	18	18	7	5	
Median ΔCt value	7.15	7.37	7.27	6.98	
Fold change to “pre“	1.00	0.86	0.92	1.13	
Average (±standard derivation)	7.12 (±0.63)	7.40 (±0.62)	7.14 (±0.44)	7.01 (±0.25)	
*p*-value to “pre“	n.d.	0.45	0.41	0.49	

The table shows peripheral blood PD-L1 mRNA expression of the pre- and postoperative time course. For each group (G1, G2, and G3) and time point (pre, post, 2 d post, 4 d post, and 10 d post), sample size (*n*), median ΔCt value, fold change (FC) to “pre”, average, and *p*-value to “pre” are provided. The FC was determined by the ΔΔCt method comparing the average ΔCt values of the different time points, starting from the pre-op value. The significant *p*-values are written in bold letters. G1: Primary tumor resection, microvascular free flap reconstruction, and selective neck dissection due to oral squamous cell carcinoma. G2: Microvascular free flap reconstruction without present malignancy. G3: Minor maxillofacial surgeries. n.d.: not determined.

## Data Availability

The data presented in this study are available in this article.

## Supplementary Materials

The following supporting information can be downloaded at: https://www.mdpi.com/article/10.3390/cancers15153755/s1, Table S1: Tumor localization and surgical procedure in G1.
