# Supplementary material for: Postoperative Changes in Systemic Immune Tolerance Following Major Oncologic versus Minor Maxillofacial Surgery
Source: Cancers (Basel). 2023 Jul 25;15(15):3755. doi: 10.3390/cancers15153755 (PMC10417560; doi:10.3390/cancers15153755)
Supplement: Supplementary file 1 [file cancers-15-03755-s001.zip › cancers-2484703-supplementary.pdf]

Table S1. Tumor localization and surgical procedure in G1.

Case	Tumor localization	Resection type	Neck Dissection level (ND)	Transplant type
1	left mandible	Mandibular box resection	ND 1-3 left	Latissmius dorsi
2	left mandible	Mandibular box resection	ND 1-3 left	Radial forearm
3	left mandible	Mandibular box resection and partial maxillary resection	ND 1-3 left	Radial forearm
4	left mandible	Mandibular continuity resection	ND 1-3 left	Radial forearm
5	tongue left, floor of mouth	Partial floor of mouth resection	ND 1-5 left, ND 1-3 right	Radial forearm
6	tongue right	Hemiglossectomy	ND 1-3 right	Radial forearm
7	pterygomanidbular, left maxilla and mandible	Mandibular continuity resection and partial maxillary resection	ND 1-3 left	Radial forearm
8	floor of mouth right, mandible right	Mandibular continuity resection	ND 1-5 right, ND 1-3 left	Latissmius dorsi
9	retromolar left	Temporary mandible split and retromolar recection	ND 1-5 left, ND 1-3 right	Radial forearm
10	floor of mouth anterior	Partial floor of mouth resection	ND 1-5 left, ND 1-3 right	Thoracodorsalis perforator
11	left mandible	Mandibular box resection	ND 1-5 left, ND 1-3 right	Radial forearm
12	floor of mouth anterior	Mandibular continuity resection and hemiglossectomy	ND 1-5 bilateral	Anterolateral thigh
13	retromolar right	Mandibular continuity resection	ND 1-3 bilateral	Fibula
14	floor of moth right	Mandibular continuity resection	ND 1-3 right	Radial forearm
15	palate right	Hemiglossectomy, floor of mouth resection, mandibular box resection	ND 1-5 right, ND 1-3 left	Anterolateral thigh
16	tongue left	Hemiglossectomy	ND 1-3 left	Latissmius dorsi
17	palate left to floor of mouth left	Mandibular continuity resection, partial maxillary resection	ND 1-3 left	Latissmius dorsi
18	retromolar left	Mandibular continuity resection	ND 1-3 left	Latissmius dorsi
19	tongue left	Hemiglossectomy	ND 1-3 left	Radial forearm
20	floor of mouth anterior	Mandibular continuity resection	ND 1-3 bilateral	Anterolateral thigh
21	retromolar right	Mandibular box resection	ND 1-3 right	Radial forearm
22	retromolar left	Mandibular box resection	ND 1-3 left	Radial forearm
23	tongue left	Hemiglossectomy	ND 1-3 left	Radial forearm

24	floor of mouth right	Partial floor of mouth resection	ND 1-3 right	Radial forearm
25	left mandible	Mandibular continuity resection	ND 1-5 bilateral	Latissmius dorsi

The table shows tumor localization, technique and extend of tumor resection, extend of Neck Dissection (ND) and the used microvascular transplant for reconstruction. Neck Dissection levels are given according to the Robbins classification.
